# Catharanthus roseus intoxication mimicking acute cholangitis

**DOI:** 10.1186/s12906-024-04441-1

**Published:** 2024-04-04

**Authors:** Yoen Young Chuah, Yeong Yeh Lee, Chu-Kuang Chou, Li-Jen Chang

**Affiliations:** 1 Division of Gastroenterology and Hepatology, Department of Internal Medicine, Ping Tung Christian Hospital, Pingtung, Taiwan; 2 Department of Nursing, Meiho University, Pingtung, Taiwan; 3 Department of Medicine, School of Medical Sciences, Universiti Sains Malaysia, Kota Bahru, Malaysia; 4 Division of Gastroenterology and Hepatology, Department of Internal Medicine, Ditmanson Medical Foundation Chia-Yi Christian Hospital, Chiayi, Taiwan; 5 Obesity center, Ditmanson Medical Foundation Chia-Yi Christian Hospital, Chiayi, Taiwan; 6 Min-Hwei Junior College of Health Care Management, Tainan, Taiwan

**Keywords:** Catharanthus roseus intoxication, Cholangitis, Jaundice, Gastric ulcers, Vinca alkaloids

## Abstract

**Background:**

Catharanthus roseus, a Madagascar native flowering plant, is known for its glossy leaves and vibrant flowers, and its medicinal significance due to its alkaloid compounds. As a source of vinblastine and vincristine used in chemotherapy, Catharanthus roseus is also employed in traditional medicine with its flower and stalks in dried form. Its toxicity can lead to various adverse effects. We report a case of Catharanthus roseus juice toxicity presenting as acute cholangitis, emphasizing the importance of healthcare providers obtaining detailed herbal supplement histories.

**Case presentation:**

A 65-year-old woman presented with abdominal pain, fever, anorexia, and lower limb numbness. Initial diagnosis of acute cholangitis was considered, but imaging excluded common bile duct stones. Further investigation revealed a history of ingesting Catharanthus roseus juice for neck pain. Laboratory findings showed leukocytosis, elevated liver enzymes, and hyperbilirubinemia. The patient developed gastric ulcers, possibly due to alkaloids in Catharanthus roseus. No bacterial growth was noted in blood cultures. The patient recovered after discontinuing the herbal extract.

**Conclusions:**

Catharanthus roseus toxicity can manifest as fever, hepatotoxicity with cholestatic jaundice, and gastric ulcers, mimicking acute cholangitis. Awareness of herbal supplement use and potential toxicities is crucial for healthcare providers to ensure prompt diagnosis and appropriate management. This case emphasizes the need for public awareness regarding the possible toxicity of therapeutic herbs and the importance of comprehensive patient histories in healthcare settings.

## Background

Catharanthus roseus is a species of flowering plant classified within the family Apocynaceae. Belonging to the genus Catharanthus, this plant is characterized by its glossy leaves and vibrant, pinwheel-like flowers. Native to Madagascar, it has been widely cultivated for its ornamental value and is also recognized for its alkaloid compounds, giving it medicinal significance in various cultures. The components of Catharanthus roseus known as vinblastine and vincristine has been widely used for chemotherapy in clinical practice [1]. Catharanthus roseus can be easily found on street in Taiwan and is accessible in traditional Chinese herbal store. It is widely used in traditional Chinese medicine to treat diabetes, gastrointestinal diseases and also used as anti-cancer drug with its flower and stalk portions in dried form for the cooking of herbal soup [2]. However, Catharanthus roseus toxicity can lead to bone marrow suppression, gastrointestinal toxicity, paresthesia, stomatitis, fever, and even cause multi-organ failure [3, 4]. We herein report a case of Catharanthus roseus juice toxicity presenting as acute cholangitis. To our best knowledge, this might be the first case of Catharanthus roseus poisoning in the literature. Our case highlights the importance of the healthcare providers in obtaining detailed patient histories of herbal or plant supplement use, and the awareness of its potential toxicities.

## Case report

**Fig. 1:**
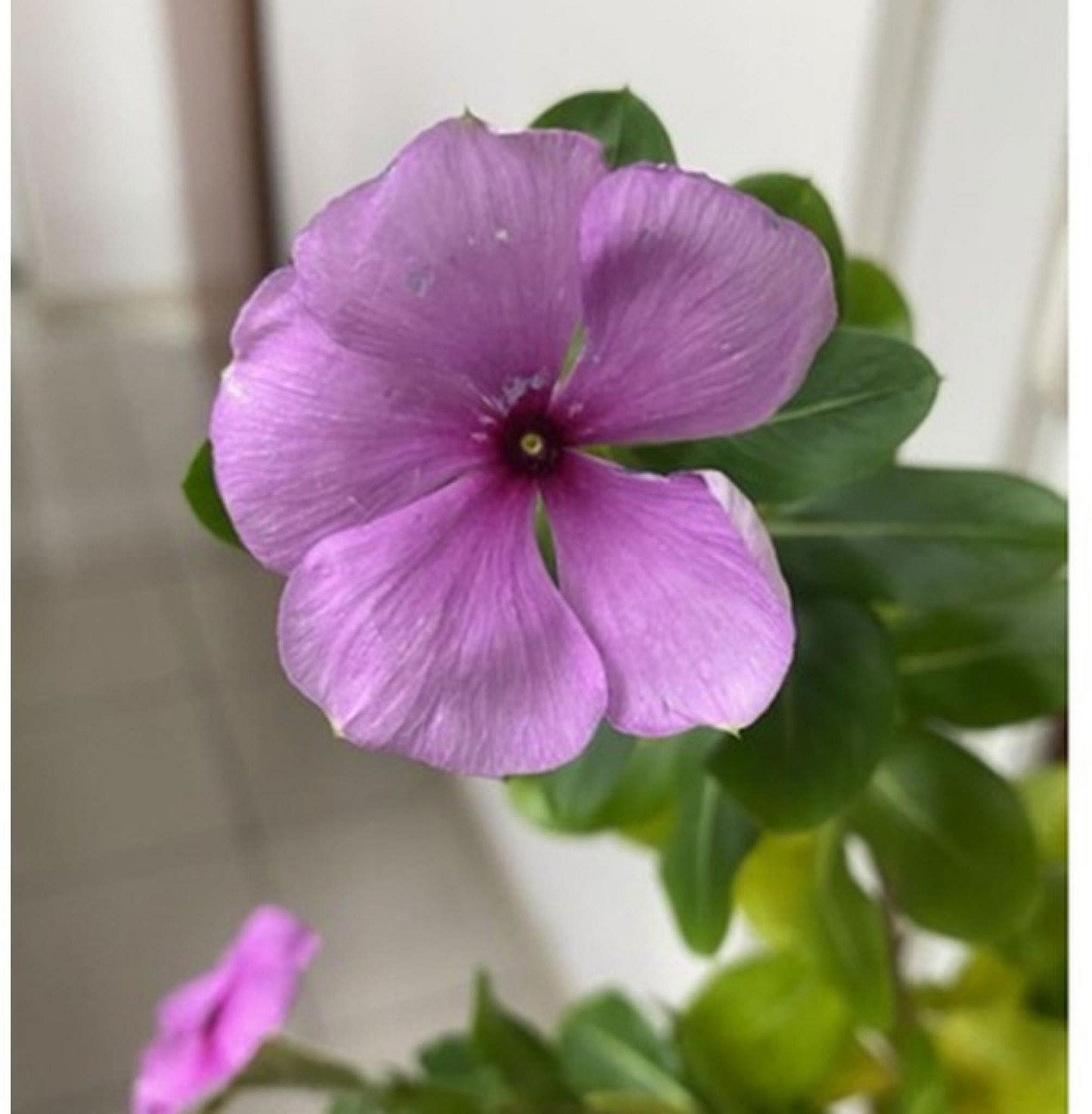
Catharanthus roseus flower photo provided by patient

**Fig. 2:**
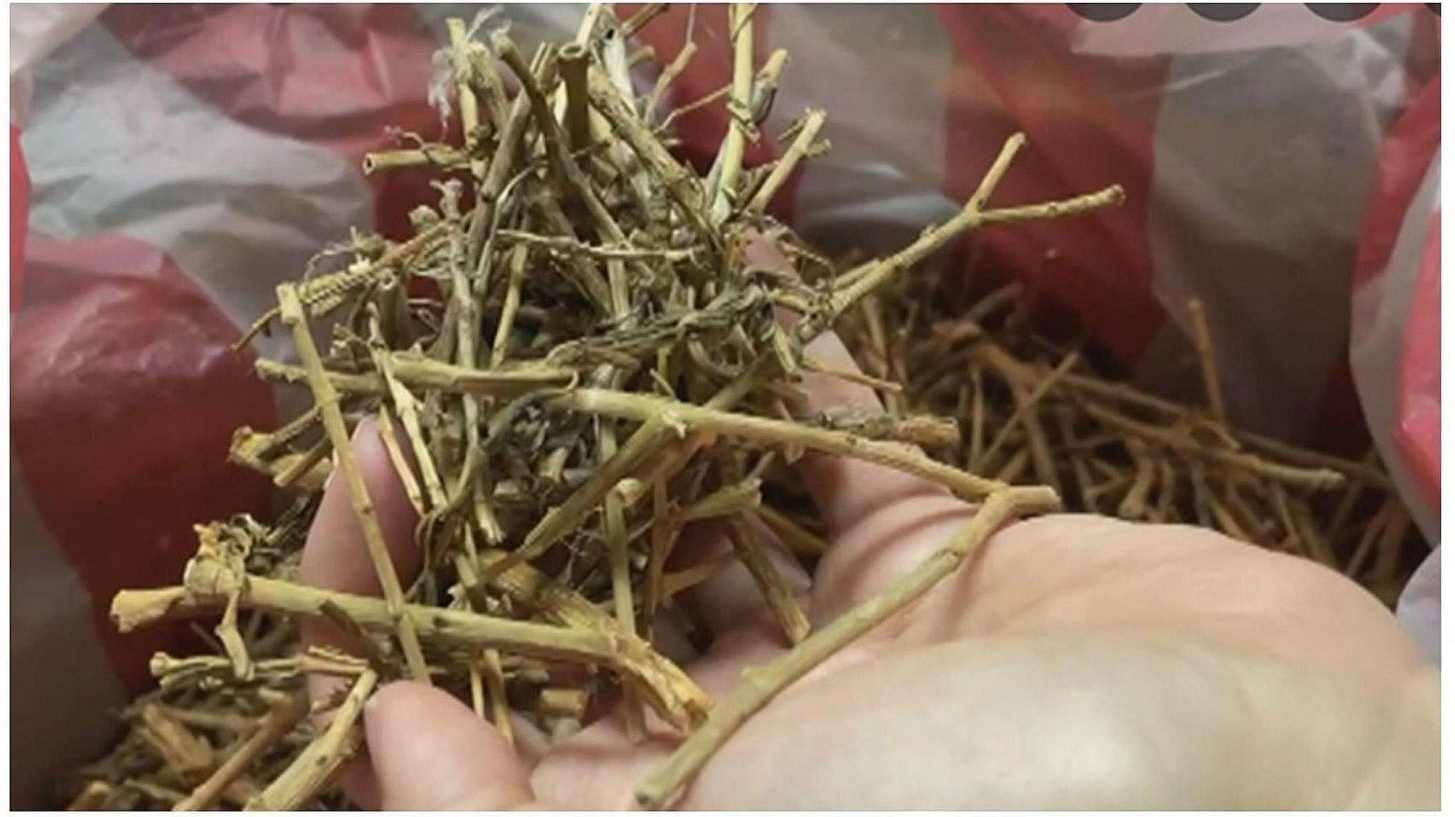
Dried flower and stalks of Catharanthus roseus photo taken at Chinese herbal store in Ping-Tung

A 65-year-old woman presented to the emergency department with a 2-day history of acute abdominal pain and fever, anorexia, and lower limb numbness. The pain was localized in the central area of the upper abdomen and was dull in nature, with a pain score of 4 out of 10. There was no radiation of pain to the other part of the body. The patient denied any associated symptoms, such as bloating, nausea, and vomiting. Her temperature, pulse, and respiratory rate were 37.2 degrees Celsius, 74 beats per minute, 19 beats per minute, and her blood pressure was 158/85 mmHg. A physical examination showed mild icteric sclera. However, hepatomegaly, right hypochondrial tenderness, features of hepatic encephalopathy, and Murphy’s sign were all negative. Sensory tests for light touch, pinprick, and vibration were performed that showed no prominent altered sensations. Differential diagnosis at this point included acute hepatitis, acute pancreatitis, peptic ulcer disease and heavy metal intoxication. Laboratory data revealed leukocytosis (WBC: 12,060/UL), elevated liver transaminases (AST: 237 U/L; ALT: 519 U/L), alkaline phosphatase (ALP: 97 U/L), gamma glutamyl-transferase (γ-GT: 213 U/L), and hyperbilirubinemia (direct bilirubin: 0.7 mg/dL, total bilirubin: 3.8 mg/dL). Activated partial thromboplastin time (APTT), D-DIMER, urea and creatinine levels, amylase and lipase were within normal limits. She was negative for hepatitis B surface antigen (HBsAg) and Anti-HCV antibody titer. Taken into account of her clinical course, acute cholangitis was highly suspected. Empirical antibiotics treatment with intravenous cefoxitin was begun at 2 g every 8 h. The patient did not disclose taking any new drugs or traditional herbal medications on the first day of her hospitalization. Contrast computed tomography of the abdomen performed at the emergency department showed no evidence of common bile duct stones. Magnetic Resonance Cholangio-Pancreatography (MRCP) of the abdomen done on the hospital day 2 showed no choledocholithiasis, and therefore the tentative diagnosis of acute cholangitis was excluded. To further investigate her abdominal pain, gastroduodenoscopy was done that revealed two active and sharp edge gastric ulcers. On further probing her history, she reported drinking juice extract of Catharanthus roseus by boiling it as a soup, about 14 g a day for 8 consecutive days (a total consumption of 112gram, Figs. 1 and 2) before her admission for treating her neck pain caused by a cervical spinal bone spur. Serum levels of heavy metals, including copper, zinc, and mercury, were all negative. The fever subsided on the fifth day of her hospitalization with discontinuation of the use cefoxitin, and her blood culture yielded no growth of bacteria. The patient was discharged on the 8th day of hospitalization and recovered smoothly thereafter with normalized liver function and bilirubin level one week after discharge. Our hypothesis was further supported by the fact that the Naranjo ADR probability scale score for the association was “probable” (score 6; Table 1).

**Table 1:** Naranjo ADR probability scale score for the association of Catharanthus roseus and its intoxication

Question		Yes	No	Do Not Know	Score
1. Are there previous conclusive reports on this reaction?		+ 1	0	0	+ 1
2. Did the adverse event appear after the suspected drug was administered?		+ 2	-1	0	+ 2
3. Did the adverse event improve when the drug was discontinued or a specific antagonist was administered?		+ 1	0	0	+ 1
4. Did the adverse event reappear when the drug was readministered?		+ 2	-1	0	0
5. Are there alternative causes that could on their own have caused the reaction?		-1	+ 2	0	+ 2
6. Did the reaction reappear when a placebo was given?		-1	+ 1	0	0
7. Was the drug detected in blood or other fluids in concentrations known to be toxic?		+ 1	0	0	0
8. Was the reaction more severe when the dose was increased or less severe when the dose was decreased?		+ 1	0	0	0
9. Did the patient have a similar reaction to the same or similar drugs in any previous exposure?		+ 1	0	0	0
10. Was the adverse event confirmed by any objective evidence?		+ 1	0	0	0
		**Total Score: 6**			

Naranjo Score ≥ 9 = Definite, 5 to 8 = Probable, 1 to 4 = Possible, ≤ 0 = Doubtful

Source: Naranjo CA, Busto U, Sellers EM, Sandor P, Ruiz I, Roberts EA, Janecek E, et al. A method for estimating the probability of adverse drug reactions. Clin Pharacol Ther. 1981;30:239–45

## Discussion

Catharanthus roseus is a raw material of anticancer drugs vinblastine, vincristine.

and vinorelbine which is widely used for chemotherapy, mainly for hematological malignancies [1]. The toxicity of Catharanthus roseus includes bone marrow suppression and gastrointestinal toxicity, similar to the toxic effects of these vinca alkaloids, but also includes paresthesia, stomatitis, fever and could lead to multi-organ failure [3, 4]. Our case presented with symptoms of lower leg numbness indicative of neurotoxicity following the ingestion of Catharanthus roseus. Alkaloids like vincristine are known to exert neurotoxic effects by interfering with microtubule formation, leading to disruption of normal cellular processes [5]. 

Hepatotoxicity is characterized by abnormalities in liver function tests and clinical signs of liver dysfunction. While hepatotoxicity associated with Catharanthus roseus is less commonly reported, it is plausible that certain alkaloids may exert adverse effects on hepatic tissues. According to one animal study in albino rats, a dose of 300 mg of Catharanthus roseus could cause biochemical and histopathological toxicity in liver, kidney and heart [6]. Similarly, hepatotoxicity is not a common concern in patients using vinca alkaloids as part of chemotherapy, even though it is metabolized by the liver and excreted in the bile. While the exact reason for this is unknown, it is possible that resting liver tissue is less susceptible to disruption of cell division.

The liver plays a crucial role in the metabolism and detoxification of various substances, and any disruption in its normal function can have systemic repercussions. In this case, it is conceivable that the alkaloids present in Catharanthus roseus may have contributed to hepatocellular injury, leading to the observed hepatotoxic effects [7]. Our case underscores the potential hepatotoxic effects of Catharanthus roseus, which, to our knowledge, has not been extensively documented in the literature and to our best understanding this might be first documented case in human. Another animal study in sheep with accidental poisoning of Catharanthus roseus were observed to have anorexia and marked increase in Activated Partial Thromboplastin Time (APTT), D-DIMER, hemoglobin, urea and creatinine levels with the decrease in cholinesterase activity and calcium levels [8]. However, relevant laboratory findings suggestive of disseminated intravascular coagulation (DIC) were not observed in our patient except for liver function derangement, which was seen in an albino rat study. Two reasons might be due: first, the metabolism of Catharanthus roseus in rats and sheep might be different from that in humans, which causes different abnormal laboratory findings; and second, the difference in dosages ingested among animals and humans might cause different kinds of toxicity.

Several compounds present in Catharanthus roseus, such as alkaloids, tannins, and glycosides, have been identified as potential contributors to gastrotoxic effects. The alkaloids vincristine and vinblastine, while effective against cancer cells, can also exert toxic effects on normal cells, including those in the gastrointestinal lining. This may lead to adverse gastrointestinal reactions when consumed inappropriately. Additionally, tannins found in the plant can bind to proteins and cause precipitation, leading to irritation and damage to the mucosal lining of the digestive tract if were ingested in high concentration [9–11]. These might be the possible reasons causing peptic ulcer in our case.

## Conclusion

Misuse of Catharanthus roseus herb may lead to hepato-toxicity with cholestatic jaundice and gastric ulcers presenting as abdominal pain with fever that mimicking acute cholangitis as like our case. Our case underscores the importance of public awareness regarding the potential toxicity of medicinal plants. Healthcare providers should be vigilant in obtaining detailed patient histories, including herbal or plant supplement use, to identify potential sources of toxicity.

## Data Availability

All data generated or analyzed during this study are included in this published article.
